# Incorporating Spectral and Directional Leaf Reflectance into Virtual Plant Models via Phong Shader Parameter Fitting

**DOI:** 10.3390/plants14172775

**Published:** 2025-09-04

**Authors:** Jens Balasus, Felix Wirth, Alexander Herzog, Tran Quoc Khanh

**Affiliations:** Laboratory of Adaptive Lighting Systems and Visual Processing, Technical University of Darmstadt, Hochschulstr. 4a, 64289 Darmstadt, Germany; wirth@lichttechnik.tu-darmstadt.de (F.W.); herzog@lichttechnik.tu-darmstadt.de (A.H.); khanh@lichttechnik.tu-darmstadt.de (T.Q.K.)

**Keywords:** leaf reflectance, spectral BRDF, virtual plant simulation, ray-tracing, GroIMP, Phong shader

## Abstract

Accurate light simulations using virtual plant models are essential for analyzing how plant structures influence the micro-light climate within canopies. Such simulations are increasingly important in applications including remote sensing, greenhouse optimization, and synthetic data generation for agricultural systems. However, many current models simplify leaf optical behavior by assuming purely diffuse reflectance, thereby neglecting the spectral and angular variability described by the bidirectional reflectance distribution function (BRDF). To address this limitation, the spectral BRDF of cucumber leaves was experimentally measured and corresponding Phong reflectance model parameters were determined for use in the GroIMP simulation environment. These parameters were optimized to replicate the angular and spectral reflectance distribution patterns and evaluated against a diffuse reflectance model. The Phong model successfully reproduced key features of the BRDF, particularly the increased diffuseness in the green and far-red spectral regions, although deviations in hemispherical reflectance emerged at high incidence angles. The resulting Phong parameters offer a practical method for incorporating wavelength- and direction-dependent reflectance into virtual plant simulations. These parameters can be adapted to other reflectance values of leaves with similar optical properties using hemispherical reflectance measurements, enabling more realistic light modeling in virtual canopies. Within a 30–60° incidence, the Phong BRDF reduced per-wavelength error relative to a diffuse baseline across all spectral regions.

## 1. Introduction

Light is a central driver of plant growth, development, and metabolism. As a result, simulating light–plant interactions has become a critical technique in plant science, supporting applications from crop optimization to remote sensing. Initial attempts to simulate light transport in plant canopies relied on classical radiative transfer models [1]. These approaches described the canopy as a homogeneous medium, averaging structural and optical properties over large volumes. However, such simplifications could not adequately represent spatial heterogeneity or angle-dependent phenomena, which are critical for realistic simulation of light–plant interactions. To overcome these limitations, ray-based methods were introduced, enabling the explicit modeling of individual plant organs and the angular distribution of light within heterogeneous canopies.

Ray-based simulations often employ Monte-Carlo ray tracing, which models light transport through stochastic sampling of the rendering equation. In contrast to radiosity models that assume diffuse energy exchange [2], Monte-Carlo methods capture directional reflectance behavior via the bidirectional reflectance distribution function (BRDF [3]), enabling simulation of angular-dependent reflectance phenomena [2].

The advent of three-dimensional (3D) virtual plant models, coupled with ray tracing techniques, has advanced the analysis of canopy light environments. These approaches have been widely applied to simulate light interception (e.g., [4,5,6]), canopy photosynthesis and light use efficiency (e.g., [7,8]) as well as responses to lighting in greenhouse systems (e.g., [9,10,11]).

In the context of plant modeling, the work by [12] demonstrated the application of quasi-Monte-Carlo path tracing to simulate both spectral and directional properties of light in virtual canopies constructed using L-systems. This approach improved convergence and facilitated biologically relevant outputs such as red:far-red ratios and localized PAR estimation.

Accurately simulating these light interactions requires a detailed understanding of the directional and wavelength-dependent properties of leaf reflectance. Early studies [13,14] identified clear deviations from purely diffuse behavior, while subsequent work linked these properties to anatomical and surface features [15,16]. Diffuse reflectance can be represented by the Lambertian model, referred to as the ‘diffuse model’ in this work. Alternatively, the physically motivated Oren-Nayar approach [17] accounts for surface roughness effects in the diffuse component.

In contrast, modeling of the specular component requires alternative formulations. The physically based Cook-Torrance model [18,19] which is grounded in microfacet theory, has also been adapted to simulate the specular reflectance of leaves [20]. In these adaptations, critical surface properties such as Fresnel effects, surface roughness and masking are included to accurately capture the spectrally dependent specular reflectance observed in plant leaves.

Building on these developments, Bousquet et al. [21] modeled the spectral BRDF of leaves, confirming the strong wavelength dependence of both diffuse and specular behavior. More recently, Roth et al. [22] included a masking term from Smith [23,24,25] and validated the model for tree leaves. In addition, Li et al. [26] introduced the PROSPECULAR model, which extends microfacet approaches by explicitly simulating multi-angular reflectance and transmittance of leaves, thereby providing an advanced physically based framework for leaf BRDF modeling. Recently, Peng and Zhu [27] introduced polarization in a microfacet-BRDF model.

Despite these advances, practical implementation of complex BRDF models remains challenging. In virtual plant modeling software such as GroIMP [28], simplified shader models, particularly the Phong model [29,30] are implemented due to their computational efficiency and ease of integration. While physically based microfacet models, such as those validated by Bousquet et al. and Roth et al. [21,22], accurately represent spectral leaf BRDF, their complexity often precludes their practical use. The need for numerous parameters and custom implementation creates a barrier to their integration into functional-structural plant models, resulting in the prevalent use of simpler diffuse-only approximations. This creates a gap between theoretical capabilities and practical applications.

To address this gap, we propose evaluating the Phong model as a pragmatic solution for integrating directional reflectance effects in plant canopy simulations. The Phong model introduces directional anisotropy using only three parameters and is natively supported in platforms like GroIMP. We thus assess whether this simplified approach can adequately capture essential optical characteristics of plant leaves, balancing realism and computational feasibility.

In this study Cucumber (*Cucumis sativus*) was selected as a model plant due to its relevance in agricultural research and favorable leaf geometry for optical measurement. Its cultivation in greenhouses makes it particularly relevant for light environment studies. Recent advances in cucumber disease detection and prediction [31] further underscore its agricultural significance. Due to its flat and large leaves, the cucumber was selected to reduce the influence of its geometry on the optical measurements. Moreover, cucumber has been established as a scientific model plant. Its geometry is well described [32] to be used in the field of virtual plants: Kahlen et al. [33] modeled leaf-phototropism, Chen et al. [7] created a photosynthesis model and in other studies [34,35] the light perception under different lighting situations was investigated. Importantly, none of the studies used spectral and angular dependent reflectance properties.

We introduce a method that fits the Phong reflectance model to experimentally measured spectral BRDF data.

Specifically, we conducted the following:

- Experimentally measured spectral BRDF of cucumber leaves across surfaces and leaf orders.
- Developed an optimization workflow to determine parameters for use with the Phong reflectance model.
- Validated spectral and angular accuracy of the fit relative to measured data.
- Demonstrated parameter scalability using hemispherical reflectance, enabling transfer to other reflectance values.

This approach offers an alternative to full BRDF modeling, allowing integration into the simulation platform GroIMP without custom shader development.

## 2. Materials and Methods

This section describes the experimental setup for measuring directional and spectral reflectance, the virtual simulation environment in GroIMP and the optimization and validation procedures used to fit the Phong shader parameters.

### 2.1. Definition and Measurement of Directional and Spectral Reflectance

**Figure 1:**
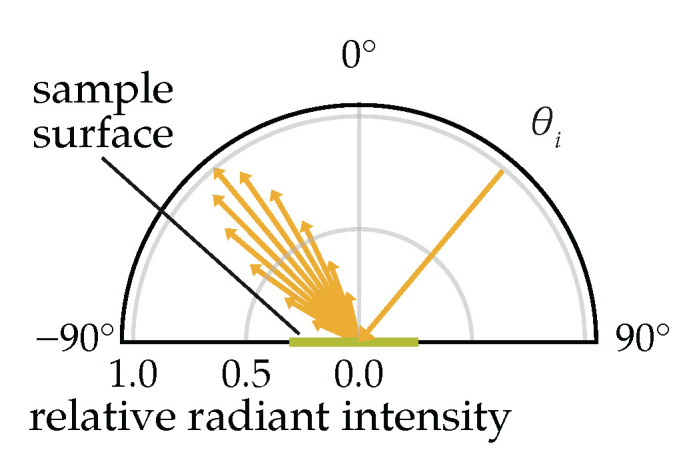
Scattering of light on a sample surface with an angle of incidence $\theta_{i}$ of $40^{\circ }$.

The directional reflectance behavior (general example shown in Figure 1) of cucumber leaves was characterized using the spectral bidirectional reflected intensity distribution function (BRIDF), derived from the BRDF formulation [3]. Unlike the BRDF, which defines reflectance per unit solid angle, the BRIDF scales reflectance by the cosine of the reflection angle ($\theta_{r}$), yielding an intuitive ray-based representation more analogous to a luminous intensity distribution curve which is widely used in lighting engineering. Assuming azimuthal isotropy of the sample surface, the BRIDF becomes a function of the angle of incidence ($\theta_{i}$), the reflection angle ($\theta_{r}$), as well as on the radiance $L_{r}$ and the irradiance $E_{e}$. For fixed $\theta_{i}$, a univariate form, the URIDF, describes the angular distribution of reflected light:

(1) $$\begin{array}{cc} f_{\mathrm{URIDF}}\left(\theta_{i}=40^{\circ },\theta_{r}\right) & =\frac{dL_{r}\left(\theta_{r},\theta_{i}=40^{\circ }\right)}{dE_{e}\left(\theta_{i}=40^{\circ }\right)}\;\cdot \;\cos\left(\theta_{r}\right) \end{array}$$

**Figure 2:**
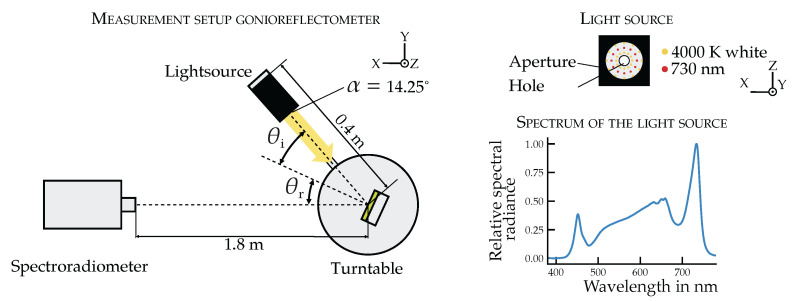
Measurement setup for measuring the BRIDF, consisting of a CS2000 spectroradiometer from Konica Minolta, the light source placed on a rotatable arm and a turntable with a sample holder (**left**). Relative spectral radiance distribution of the light source used for the BRIDF measurement, composed of white 4000 K LEDs and 730 nm LEDs (**right**), measured with the CS2000 on a white standard.

The BRIDF is measured using a spectral gonioreflectometer based on the setup of Li et al. [36]. This setup includes (1) a motorized turntable with a sample holder (rotation axis: $\theta_{r}$), (2) a movable light source arm (rotation axis: $\theta_{i}$), and (3) a fixed-position spectroradiometer (CS2000, Konica Minolta, Langenhagen, Germany). The measurement setup allows for the fully automated acquisition of reflected spectral radiance across a wide range of angles and wavelengths (see Figure 2). In this process, the spectroradiometer records the spectral radiance reflected from the sample, which is subsequently referenced to the radiance of a calibrated white standard.

The light source is composed of a Luxeon TX white LED (4000 K) and supplementary 730 nm LEDs (ColorLine 2835, Lumileds, Schiphol, Netherlands), to enhance sensitivity in the far-red spectral range, which is physiologically relevant for plants. The LEDs are concentrically arranged on a printed circuit board with a central aperture to allow near-normal incidence (Figure 2, right). The source is collimated by an aperture, resulting in a defined beam angle of 14.25°.

The spectroradiometer operates with a 0.2° aperture angle, corresponding to a 6.2mm spot size on the leaf surface. This angle provides adequate spatial averaging over leaf microstructure while avoiding edge overlap at oblique angles.

Each measurement series was normalized with a white standard at $\theta_{i}=\theta_{r}=0^{\circ }$. Because the source intensity is constant, irradiance at the sample depends only on $\theta_{i}$. The BRIDF, as defined by [3], is computed using the relationship E=L·π for diffuse surfaces:

(2) $$f_{\mathrm{BRIDF}}\left(\theta_{i},\theta_{r},\lambda \right)=\frac{L_{e,\mathrm{probe}}\left(\theta_{r},\theta_{i},\lambda \right)}{L_{\mathrm{WST}}\left(\lambda \right)\;\cdot \;\cos\left(\theta_{i}\right)\;\cdot \;\pi }\;\cdot \;\cos\left(\theta_{r}\right)$$

where $L_{e,\mathrm{probe}}\left(\lambda \right)$ is the radiance from the sample and $L_{\mathrm{WST}}\left(\lambda \right)$ the radiance from the white standard.

### 2.2. Measurement of Spectral Reflectance and Transmittance

To complement the angular BRIDF data with absolute reflectance and transmittance values, hemispherical spectral measurements were conducted using an integrating sphere setup. These values are required to scale the relative distribution from the BRIDF to hemispherical reflection and transmission.

**Figure 3:**
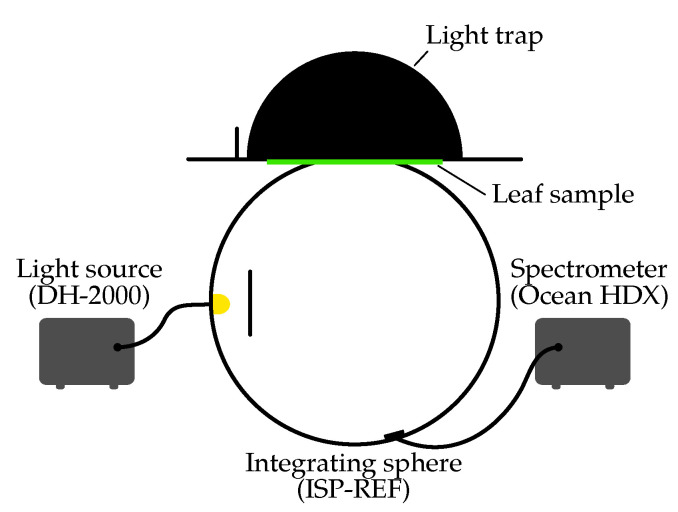
Schematic illustration of the measurement setup that was utilized to measure the spectral reflectance of leaves. A deuterium-halogen lamp is utilized to illuminate the leaf sample, which is maintained at the port of an integrating sphere. The light that has been reflected is collected by the sphere and measured by a fiber-coupled spectrometer.

The measurements were conducted using an ISP-REF integrating sphere (Ocean Optics, Orlando, FL, USA) coupled to an Ocean HDX spectrometer (Ocean Optics, Orlando, FL, USA) (Figure 3). The integrating sphere facilitates the quantification of the total reflected spectral radiant flux from the sample. By referencing the measured flux to that obtained from a calibrated white standard, the spectral reflectance of the sample can be determined.

A deuterium–halogen tungsten lamp (DH-2000, Ocean Optics) served as the primary light source for reflection measurements. During these measurements, the source beam was coupled directly into the sphere, with the sample placed at the port.

For transmittance measurements, the primary lamp was turned off and a secondary LED-based light source was used to uniformly illuminate the sample from behind. This source included a CRI > 90 white LED (Luxeon TX, 4000 K, LUMILEDS, Schiphol, The Netherlands), supplemented by 730 nm (ColorLine 2835) and 470 nm (NF2E757HT-F1, Nichia, Anan, Japan) LEDs. All LEDs were driven by independent constant-current sources. Prior to measurements, the LEDs underwent a 200-h burn-in period [37,38] followed by 4 h of thermal stabilization to ensure emission stability in the beginning of each measurement cycle.

This setup provides the absolute spectral hemispherical reflectance and transmittance of the leaf samples, which are later used to scale the relative BRIDF measurements. While the BRIDF captures the angular distribution pattern of reflectance, only the hemispherical measurements allow for calibration to absolute reflectance values. Together, both datasets enable realistic parameterization of the Phong model.

### 2.3. Determination of Phong Parameters

To incorporate the BRIDF into ray tracing simulations in GroIMP, the PhongShader is used [39].

**Figure 4:**
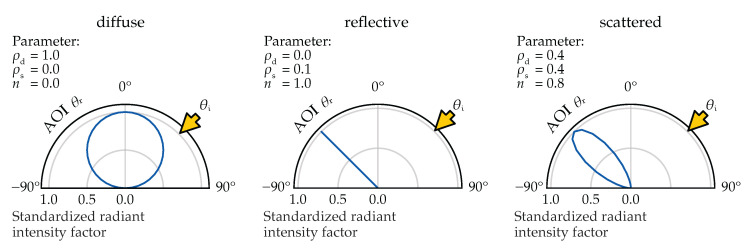
BRIDF for different parameter configurations determined using the Equation derived from Blinn’s work [30]. The equation and its derivation can be found in [40] (pp. 39–40). The diffuse BRIDF is the standard case used in virtual plant lighting simulations.

Figure 4 illustrates how different combinations of these parameters influence the resulting BRIDF, demonstrating the range from purely diffuse to specular reflection patterns. Since there is no analytical inversion of the BRIDF to shader parameters, the best-fit values must be determined empirically.

#### Virtual Gonioreflectometer in GroIMP

**Figure 5:**
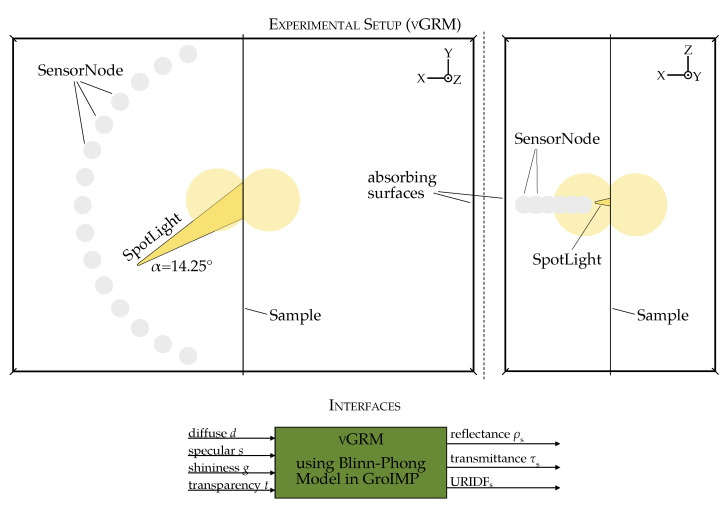
Sketched structure of the virtual gonioreflectometer. The proportions are not to scale. Top view (**upper left**) and view from the side (**upper right**). Interfaces with inputs and outputs of the virtual gonioreflectometer (**bottom**).

A virtual gonioreflectometer model (vGRM) was implemented in GroIMP to simulate the BRIDF as computed by the PhongShader and compare it with measured values (Figure 5). This setup mimics the physical gonioreflectometer and consists of: (1) a SpotLight source with a defined angular aperture ($\alpha =14.25^{\circ }$), (2) a semi-circular array of SensorNodes to capture reflected irradiance and (3) a square sample surface with adjustable reflectance.

The geometry is enclosed in absorbing walls to eliminate stray light and isolate directional reflectance. To reduce computation error, all simulations were run with 10 million rays per configuration. A 1:30 distance-to-sensor-diameter ratio was found optimal to achieve an angular resolution of 2° without overlap.

The parameters *d*, *s* and *g* in GroIMP’s PhongShader correspond to $\rho_{d}$, $\rho_{s}$ and *n* of the common Phong model, respectively. The shininess exponent is normalized in GroIMP to the range [0, 1], unlike the original Phong model where *n* spans higher integers. These parameters are passed to the shader via input interfaces and the resulting URIDF, hemispherical reflectance, and transmittance are recorded as outputs from each simulation.

The vGRM includes the following interfaces:

- Input Parameters: Adjustable Phong parameters (diffuse, specular, shininess exponent and transparency) to control the optical properties of the sample.
- Output results: The hemispherical reflectance, hemispherical transmittance and the URIDF for the specified Phong parameters.

The vGRM can be controlled programmatically via external environments such as Python (version 3.9.0). This interface allows integration with optimization routines, enabling automated parameter tuning and convergence tracking during the fitting process.

### 2.4. Optimization Process

The Phong parameters were determined using a Python (version 3.9.0) -based optimization pipeline built on the GroPy library (version 0.0.3 (https://gitlab.com/grogra/groimp-utils/pythonapilibrary (accessed on 8 July 2025)). The vGRM was used to generate URIDFs for each candidate parameter set, which were then compared to the measured URIDFs using a non-linear least-squares minimization approach implemented via the LMFIT library [41]. This algorithm was previously used in [22] for fitting BRDF models.

**Figure 6:**
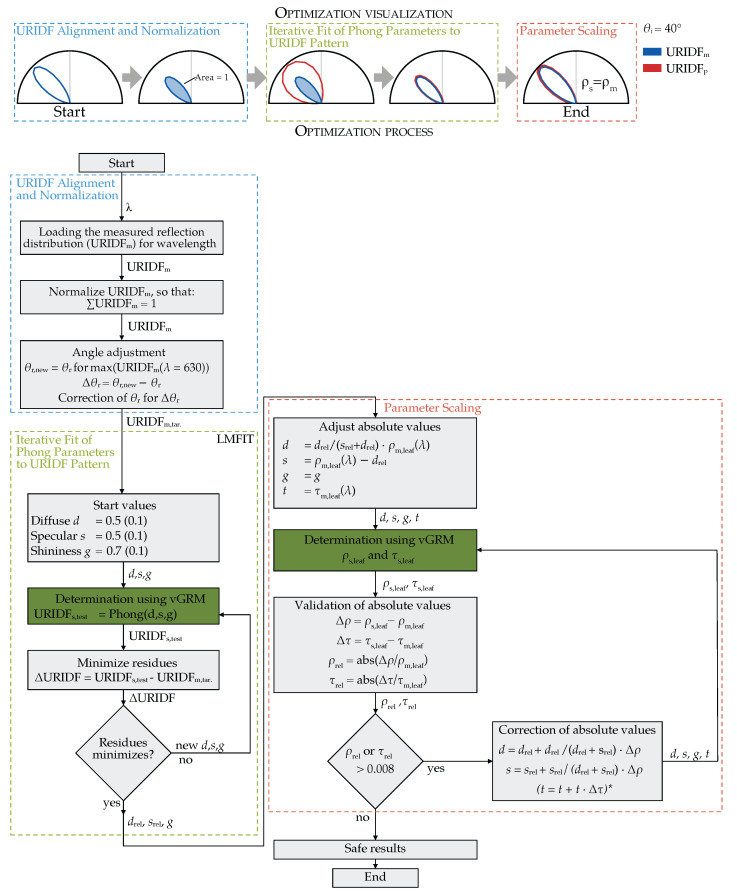
Parameter optimization procedure to determine the correct parameters to be used within GroIMP. The library [41] serves as the LMFIT algorithm. The optimization process is structured as follows: operations within the green filled box are executed in GroIMP, while all other steps are performed in Python. (version 3.9.0) ${\mathrm{URIDF}}_{m}$ represents the measured URIDF, while ${\mathrm{URIDF}}_{p}$ the calculated one using the PhongShader. $\tau_{s,\mathrm{leaf}}$ is the simulated transmittance, while $\rho_{s,\mathrm{leaf}}$ is the simulated reflectance. * The transmittance was also checked, but there was no deviation in any case.

The optimization process consisted of three sequential stages (as shown in Figure 6): (1) Preprocessing and normalization of measured URIDFs, (2) iterative fitting of the Phong parameters to match the distribution pattern, and (3) scaling the fitted parameters to align with hemispherical reflectance and transmittance measurements.

#### 2.4.1. URIDF Alignment and Normalization

Prior to fitting, each measured spectral URIDF was normalized so that the integral of the curve equals one. This normalization ensures consistency across wavelengths and enables separation of the reflectance distribution pattern from its absolute magnitude.

Due to the cosine weighting in the BRIDF definition, the angular peak of the reflection shifts systematically away from the specular direction. This effect was quantified using vGRM simulations at varying angles of incidence: e.g., the peak was displaced by approximately −2°, −3°, and −6° for $\theta_{i}$ = 20°, 40°, and 60°, respectively.

To align the simulated and measured curves, the measured URIDF was angularly shifted by the offset determined from the 630 nm peak. This correction also compensates for non-ideal alignment of the leaf surface in the physical measurement setup (e.g., curvature-induced tilt).

#### 2.4.2. Iterative Fit of Phong Parameters to URIDF Pattern

Using the corrected and normalized URIDF, the Phong model parameters (*d*, *s*, *g*) were iteratively adjusted to minimize the residual between the simulated and measured distribution patterns. Optimization was performed using the LMFIT algorithm with default settings.

Initial values for *d* and *s* were set to 0.5, while *g* was initialized at 0.7, based on preliminary performance. At each iteration, the vGRM returned the simulated URIDF. The residual vector (${\mathrm{URIDF}}_{\mathrm{sim}}$−${\mathrm{URIDF}}_{\mathrm{meas}}$) was used to assess convergence. Once the pattern fit reached a defined threshold, the parameters were scaled to match hemispherical reflectance.

#### 2.4.3. Scaling Parameters to Hemispherical Reflectance

To match the absolute reflectance of real leaves, the fitted shader parameters were scaled using measured hemispherical reflectance ($\rho_{m}$) and transmittance ($\tau_{m}$) data. Assuming that the shape of the URIDF is preserved, the diffuse and specular components were rescaled proportionally while keeping their ratio constant. The PhongShader’s transmittance parameter was set directly to match $\tau_{m}$, assuming a diffuse-only transmission model.

Each adjusted parameter set was re-validated in the vGRM. If the simulated values deviated by more than 0.8% from the measurements, correction parameters were calculated and re-evaluated. The tolerance of 0.8% for the deviation between simulated and measured hemispheric reflectance was determined empirically during preliminary tests. Adjustments typically were well below 1%. The threshold of 0.8% was therefore established not as a rigid target. The highest deviation in the final dataset after correction was 0.35%, with 95% of the data below 0.21%.

### 2.5. Hemispherical Integrated Reflectance Under Diffuse Illumination

To further validate hemispheric reflectance behavior under diffuse lighting environments, an additional experiment was conducted using diffuse illumination in the vGRM. Hemispheric illumination was discretized using nine zenith segments (10° to 90°) and twelve azimuth segments (0° to 330°), totaling 108 directional light sources. Parameter sweeps with constant theoretical reflectance but varying specular fractions were performed to assess potential implementation-specific biases in the GroIMP Phong shader.

### 2.6. Data Acquisition and Validation Criteria

To minimize measurement time and reduce dehydration effects during data acquisition, three angular datasets were collected per leaf: one for parameter fitting and two for validation. A summary of the measured data is given in Table 1.

The main dataset spanned reflection angles from −75° to 75° in 5° increments, with lower angular resolution (13°) around the fixed light source angle of 40°. Two validation datasets were acquired at incidence angles of 30° and 60°, using reflection angles from −70° to 70° in 10° increments. This asymmetric sampling around the main dataset enabled validation across both smaller and larger incidence deviations.

Each dataset required approximately 30 min to measure. Both the adaxial and abaxial leaf surfaces were measured for each leaf order, where leaf order increases from the oldest (lowest order) to the youngest leaves.

#### Challenges and Limitations in Data Collection

The measurement setup is highly sensitive to angular deviations and even minor surface curvature or misalignment of the leaf can cause systematic offsets between the intended and actual reflection angles, particularly in the case of specular reflections.

Another limiting factor was the aperture angle of the spectroradiometer. A larger angular aperture would average over a broader leaf area, thereby reducing the influence of anatomical heterogeneity such as veins or surface curvature. However, if the aperture is too small, the measurement becomes overly sensitive to localized anatomical features, which can distort the reflectance profile. Based on preliminary tests, an aperture angle of 0.2° was selected as a trade-off: Small enough to maintain angular resolution, but large enough to reduce the impact of local variation. At extreme reflection angles (>75°), this aperture occasionally caused the measurement spot to extend beyond the leaf surface, introducing minor edge effects.

### 2.7. Validation Criteria: URIDF Distribution Pattern

The validity of the fitted Phong parameters (*d*, *s*, *g*) was assessed using the following criteria, which focus on the fidelity of the simulated URIDF relative to the measured data:

- The simulated URIDF must reproduce the shape of the measured distribution.
- The distribution pattern should remain consistent across different incidence angles (enabling generalization to BRIDF).
- The relative amplitudes at different angles of incidence should match measured data.
- The total reflectance should remain invariant with respect to the angle of incidence.

Additionally, we tested the hypothesis that the normalized root mean square error (NRMSE) for the PhongShader fit is lower than for a purely diffuse reflectance assumption.

The normalized root mean square error (NRMSE) quantifies distribution pattern deviation and is defined as

(3) $$\mathrm{NRMSE}\left(\lambda \right)=\frac{\sqrt{\frac{1}{n}\sum_{i=1}^{n}{\left({\mathrm{URIDF}}_{p}\left(\lambda \right)-{\mathrm{URIDF}}_{m}\left(\lambda \right)\right)}^{2}}}{\bar{{\mathrm{URIDF}}_{m}}\left(\lambda \right)}$$

where ${\mathrm{URIDF}}_{p}\left(\lambda \right)$ is simulated using the PhongShader in GroIMP and ${\mathrm{URIDF}}_{m}\left(\lambda \right)$ is the measured URIDF. This error measure is normalized to the mean value of the measured spectral URIDF, allowing direct comparison across different measurements and wavelengths.

#### 2.7.1. Validation at Multiple Incidence Angles

To assess the robustness of the fitted Phong parameters across different angles of incidence, we computed the NRMSE between simulated and measured URIDFs at validation angles (30° and 60°), normalized to their respective maxima. This allowed us to test whether the distribution pattern remains consistent when using parameters fitted at 40°:

(4) $${\mathrm{NRMSE}}_{p}\left(\lambda \right)=\frac{\sqrt{\frac{1}{n}\sum_{1}^{n}{\left(\frac{{\mathrm{URIDF}}_{p}\left(\lambda \right)}{{\mathrm{URIDF}}_{p},\max}-\frac{{\mathrm{URIDF}}_{m}\left(\lambda \right)}{{\mathrm{URIDF}}_{m},\max}\right)}^{2}}}{\frac{1}{n}\sum_{1}^{n}\frac{{\mathrm{URIDF}}_{m}\left(\lambda \right)}{{\mathrm{URIDF}}_{m},\max}}$$

#### 2.7.2. Diffuse Baseline Comparison

As a baseline comparison, we evaluated the NRMSE between the measured URIDF and a purely diffuse URIDF model (Lambertian distribution), scaled to match the peak intensity. This provides a reference to quantify the improvement gained by using the Phong-based BRIDF:

(5) $${\mathrm{NRMSE}}_{d}\left(\lambda \right)=\frac{\sqrt{\frac{1}{n}\sum_{1}^{n}{\left({\mathrm{URIDF}}_{d}\left(\lambda \right)-\frac{{\mathrm{URIDF}}_{m}\left(\lambda \right)}{{\mathrm{URIDF}}_{m},\max}\right)}^{2}}}{\frac{1}{n}\sum_{1}^{n}\frac{{\mathrm{URIDF}}_{m}\left(\lambda \right)}{{\mathrm{URIDF}}_{m,\max}}}$$

where ${\mathrm{URIDF}}_{d}\left(\lambda \right)$ denotes the diffuse URIDF.

#### 2.7.3. Amplitude Ratios Across Incidence Angles

To evaluate whether the relative magnitude of reflectance is preserved across angles of incidence, we computed the amplitude ratio of the URIDF maxima at 30° and 60° with respect to the main dataset at 40°:

(6) $$r\left(\lambda \right)=\frac{{\mathrm{URIDF}}_{m,\max,\theta_{i}=30}\left(\lambda \right)}{{\mathrm{URIDF}}_{m,\max,\theta_{i}=40}\left(\lambda \right)}$$

With the maximum of the URIDF of the main data set ${\mathrm{URIDF}}_{m,\max,\theta_{i}=40}$ and that of the validation data set ${\mathrm{URIDF}}_{m,\max,\theta_{i}=30}$. A similar ratio was calculated for the 60° dataset.

These ratios allow for the assessment of whether the angular dependence of reflectance magnitude is captured by the fitted Phong model, which is a key requirement for spectral light balance simulations.

#### 2.7.4. Statistical Analysis

To assess whether the Phong model yields a reliably lower error than the diffuse model, a paired per-wavelength difference in NRMSE was calculated:

(7) $$\Delta \mathrm{NRMSE}\left(\lambda \right)\;=\;{\mathrm{NRMSE}}_{\mathrm{diffuse}}\left(\lambda \right)\;-\;{\mathrm{NRMSE}}_{\mathrm{Phong}}\left(\lambda \right)$$

so that ΔNRMSE(λ)>0 indicates an improvement for the Phong model. For each wavelength and for each combination of leaf side and incidence angle, the null hypothesis $H_{0}:\;\mu_{\Delta \mathrm{NRMSE}(\lambda )}=0$ was tested using a two-sided paired *t*-test across the five leaves (one per order).

#### 2.7.5. Total Reflectance Independence of Angle-of-Incidence

Finally, to evaluate the physical plausibility of the fitted parameters, we tested whether the total simulated reflectance remains invariant under changes in the angle of incidence. This was done by simulating reflectance at 30° and 60° and comparing it to the 40° reference value. The deviation was expressed as a percentage error:

(8) $$\Delta \rho \left(\theta_{i}\right)=\frac{\rho \left(\theta_{i}\right)-\rho \left(40^{\circ }\right)}{\rho (40^{\circ })}\;\cdot \;100$$

Angle-invariance is a desirable property for reflectance models used in radiative transfer and canopy-scale energy balance simulations.

## 3. Results

Preliminary tests showed no systematic variation in the BRIDFs between individual cucumber plants. To reduce complexity, a representative plant was selected, whose measurements were used throughout the validation of the fitting and simulation pipeline.

Using the vGRM-based optimization, the PhongShader parameters were fitted individually for each wavelength and leaf surface. The resulting trends in diffuse, specular and shininess parameters provide insight into the spectral behavior of the cucumber leaves BRIDF.

### 3.1. Wavelength-Dependent Parameters

**Figure 7:**
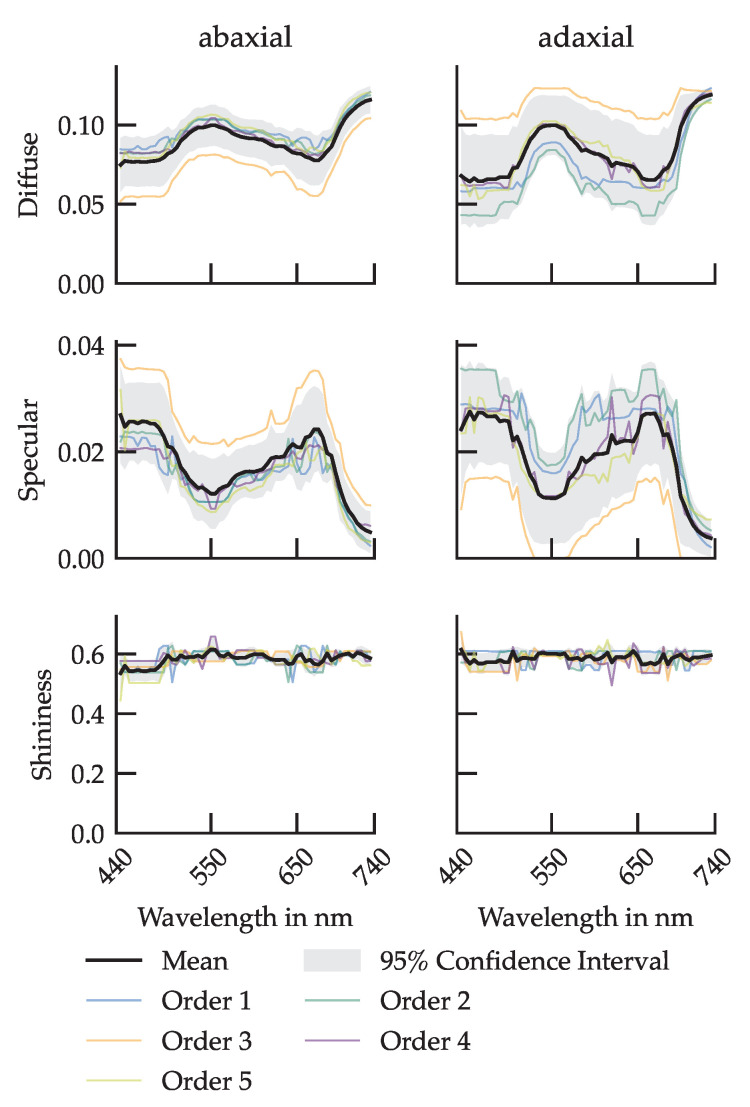
Determined spectral Phong parameters in GroIMP for the abaxial (**left**) and adaxial (**right**) leaf surfaces. Diffuse, specular and shininess are the parameters used by the Phong shader in GroIMP.

Figure 7 presents the fitted spectral Phong parameters for the adaxial and abaxial surfaces of cucumber leaves. The diffuse component (*d*) shows a consistent spectral pattern on both surfaces, with increased reflectance in the green (550 nm) and far-red (>700 nm) regions and minima around blue (450 nm) and red (670 nm) chlorophyll absorption bands. However, this spectral modulation is more pronounced on the adaxial side, where interleaf variation is also greater.

In contrast, the abaxial surface exhibits a more uniform diffuse response across leaf orders and wavelengths, with a generally flatter spectral profile.

The specular parameter (*s*) shows complementary behavior. It is generally lower in the green and far-red spectral regions, consistent with increased diffuse scattering in these bands. On the adaxial side, specular reflectance varies more strongly with leaf order. Meanwhile, the shininess parameter (*g*), which controls the angular sharpness of specular reflection, remains approximately constant across wavelengths and orders for both surfaces.

Notably, leaf order 3 deviates from these trends, especially on the adaxial surface. This order exhibits a lower diffuse and higher specular reflectance than neighboring leaves.

### 3.2. Wavelength Dependence of the Specular Reflectance Fraction

**Figure 8:**
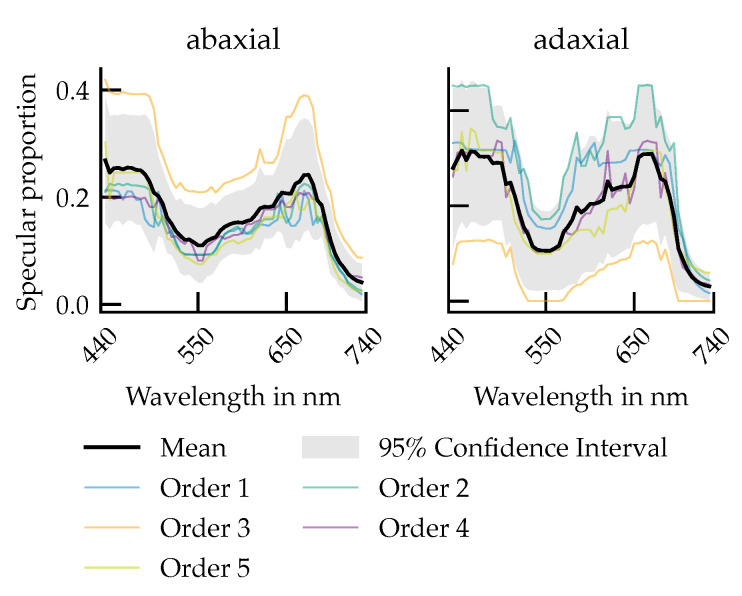
Specular proportion as a function of the wavelength for the abaxial leaf side (**left**) and the adaxial leaf side (**right**).

The proportion of specular reflectance $\rho_{s}$, calculated as the ratio

(9) $$p_{s}=\frac{s}{d+s}$$

provides a wavelength-dependent view of how sharply directed the reflected light is for each leaf surface (Figure 8). On the abaxial side, $\rho_{s}$ ranges from 20% to 40% in the blue and red spectral regions (450 nm and 670 nm), decreases to 10% from 20% in the green (550 nm) and peaks modestly again above 700 nm.

On the adaxial side, the overall spectral modulation is stronger and shows greater inter-leaf variability. In particular, leaves of order 1, 2, 4, and 5 exhibit higher specular proportions in the blue and red bands (30%), while maintaining lower values in the green and far-red. In contrast, leaf order 3 again deviates.

### 3.3. Error Analysis: Transferability and Improvement over Diffuse Models

#### 3.3.1. Transferability of Phong Parameters Across Incidence Angles

**Figure 9:**
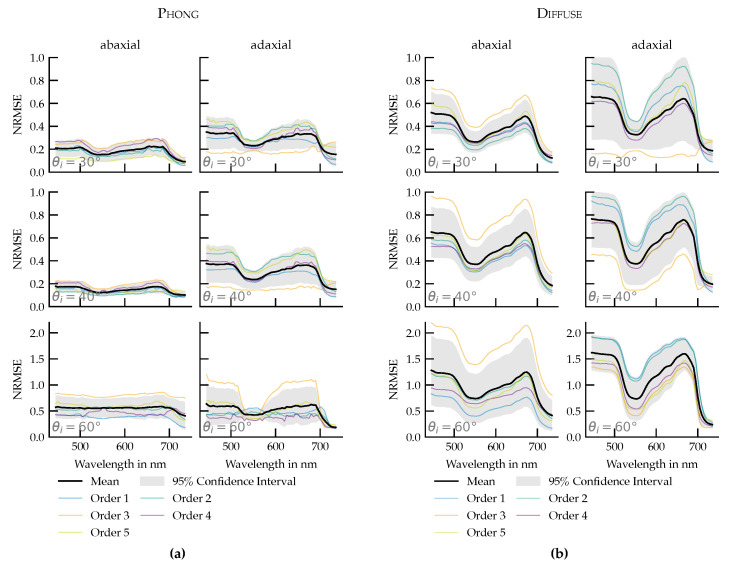
(**a**) Normalized root mean square error (NRMSE) between Phong model-simulated URIDFs and measured spectral URIDFs for the abaxial (**left**) and adaxial (**right**) leaf surfaces. (**b**) NRMSE between the measured spectral URIDFs and a purely diffuse reflection model, scaled to match the peak intensity for the abaxial (**left**) and adaxial (**right**) leaf surfaces. In both subfigures, parameters fitted at 40° were applied without refitting to 30° and 60°.

The Phong parameters were optimized using URIDF measurements at an incidence angle of 40°. To assess their transferability, the same parameters were applied to simulate URIDFs at 30° and 60° and compared to corresponding measured data. The normalized root mean square error (NRMSE) was calculated for each case (Figure 9a).

The results show that the Phong model retains high accuracy at 30°, with NRMSE values comparable to those at 40°. For both the abaxial and adaxial surfaces, differences were minor. At 60°, however, the NRMSE increased on both sides, approximately doubling on the abaxial surface. Across all datasets, adaxial surfaces showed higher NRMSE than abaxial surfaces.

#### 3.3.2. Reference Comparison: Measured URIDFs vs. Diffuse Reflection

To isolate the modeling improvement offered by BRIDF-aware fitting, a second analysis compared the measured URIDFs to a purely diffuse reflection model, scaled to match each measurement’s peak value. This benchmark captures the error introduced by ignoring angular anisotropy in reflectance (Figure 9b). Importantly, this comparison was made against the measured URIDF, not the Phong fit. It therefore reflects the fundamental discrepancy between real leaf reflectance and an isotropic (direction-independent) model, not an error in the shader fitting process.

In most cases, the NRMSE was halved. At steeper incidence angles (60°), the overall errors rose in both models, but the Phong-based simulations still outperformed the diffuse baseline.

### 3.4. Per-Wavelength Error Reduction: Phong vs. Diffuse

A quantitative comparison of the model errors reveals that the per-wavelength difference, ΔNRMSE(λ), is consistently positive across both leaf sides and all three incidence angles. This indicates a systematically lower NRMSE for the Phong model compared to the diffuse model over the measured spectrum. The magnitude of this difference is dependent on the angle of incidence, with the largest error reduction observed at 60°, followed by 40° and 30°. The spectral trend of ΔNRMSE(λ) is more uniform on the abaxial leaf side, whereas the adaxial side exhibits greater spectral variation with distinct regions of significant improvement.

**Figure 10:**
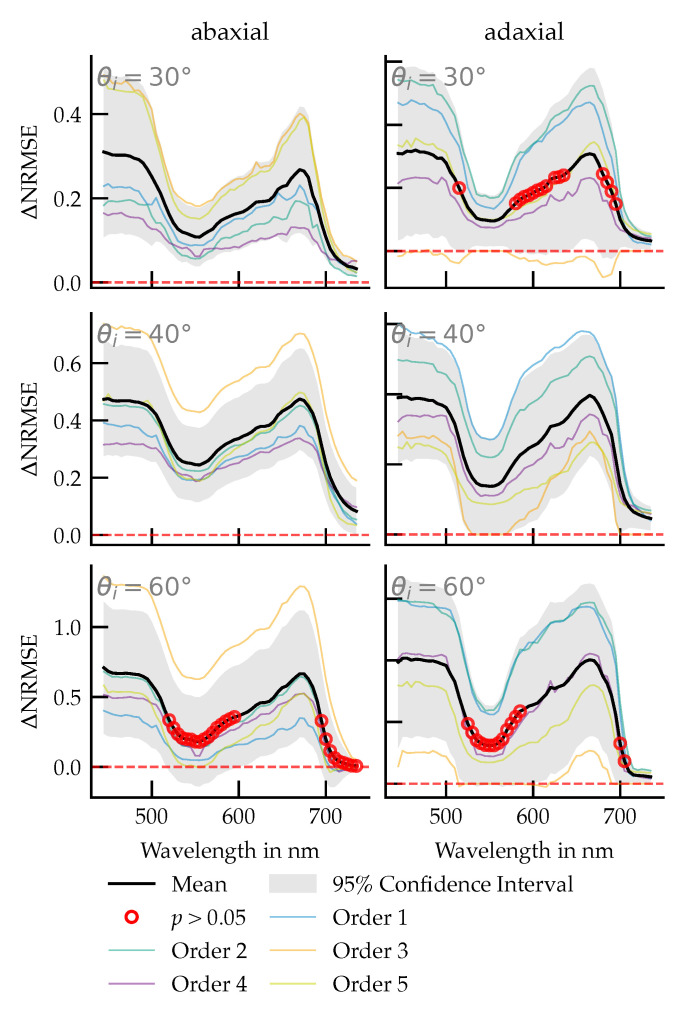
Per-wavelength difference in normalized error between the diffuse and Phong models for both leaf sides (columns) and angles of incidence (rows). Thin colored lines show individual leaves (Orders 1–5). The thick black line is the mean across leaves, the grey envelope depicts the 95% confidence interval of the mean. The red dashed horizontal line marks Δ=0. Red open circles indicate bins with non-significant differences (paired *t*-test against zero, unadjusted p≥0.05). Positive values imply lower NRMSE for the Phong model.

To assess the statistical significance of these observations, pointwise paired *t*-tests (n=5 leaves) were conducted for each wavelength (Figure 10). The results confirm that ΔNRMSE(λ) is significantly greater than zero across extended, contiguous spectral regions for all tested conditions (p<0.05). Wavelengths where the difference was not statistically significant (p≥0.05, indicated by red circles) were confined to narrow spectral bands. No statistically significant negative values for ΔNRMSE(λ) were found in any scenario, demonstrating that the diffuse model did not outperform the Phong model under any of the tested conditions.

### 3.5. Reflectance Stability Across Angles of Incidence

To assess the angular robustness of the Phong model, two complementary analyses were conducted: one focused on the spectral modulation of reflectance maxima with changing light incidence angle and the other on the total reflectance conservation across angles.

#### 3.5.1. Angular Modulation of URIDF Amplitudes

**Figure 11:**
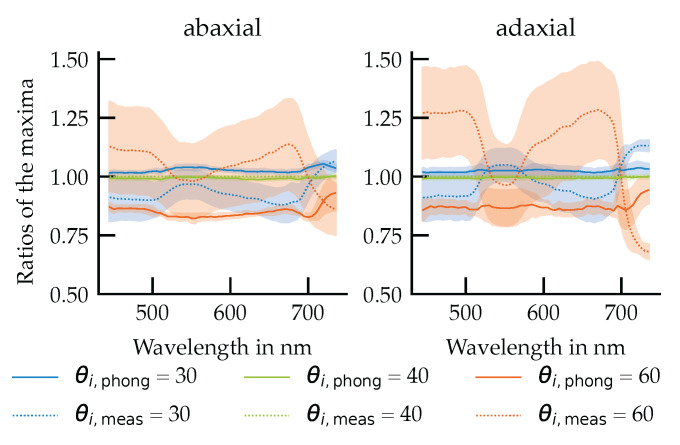
Calculated ratios of the maxima in the test data sets and the main data sets as a function of wavelength. The mean values and standard deviation are shown. The measured data set is shown as a dotted line and the modeled data set as a solid line.

While previous sections focused on the normalized URIDF distribution pattern, this analysis evaluates whether the absolute reflection maxima vary consistently with angle of incidence and whether this behavior is captured by the Phong model (Figure 11).

The results reveal a clear spectral dependence of the measured reflection maxima. At 30° and 40° incidence angles, the normalized amplitudes are slightly below unity, with values approaching unity in the green (550 nm) and far-red (>700 nm) spectral regions, where diffuse scattering dominates. At 60° incidence, the amplitudes exceed unity and exhibit an inverted spectral pattern, with higher maxima observed in the blue (450 nm) and red (670 nm) regions.

In contrast, the Phong model exhibits a more uniform spectral behavior, with reflection maxima decreasing consistently as the angle of incidence increases, largely independent of wavelength. At 60° incidence, the model underestimates the reflection maxima, with deviations in the amplitude ratios reaching up to 0.4, particularly on the adaxial surface.

#### 3.5.2. Total Reflectance Deviation with Angle

Beyond angular intensity, the model’s ability to conserve total reflected energy across different incidence angles is critical for simulating photosynthetic light absorption and canopy-level energy balances. To evaluate this, the total reflectance simulated in GroIMP at incidence angles ranging from 0° to 85° was compared to a reference value at 40° (Table 2).

Deviations were minor (<1%) at angles up to 30° and remained within ±2% at angles up to 60°, suggesting good performance within the most common incidence range in plant canopies. However, at steeper angles, deviations increased progressively, reaching −5.7% at 85%. This decline reflects the limitations of the Phong shader under grazing incidence conditions, where changes in projected area, specular sharpness and sampling density introduce increasing error.

Despite this, the mean deviation across all tested angles was 1.89%, confirming that the Phong model is reasonably stable across a broad range of conditions which is sufficient for most practical applications in ray-based plant simulations.

#### 3.5.3. Total Reflectance Deviation with Specular Proportion for Diffuse Illumination

To assess the energy conservation of the GroIMP Phong shader under diffuse illumination, a systematic test was performed. The results, presented in Table 3, reveal a systematic discrepancy between the constant theoretical reflectance (0.20) and the simulated output, which is strongly dependent on the specular fraction.

For a purely diffuse surface (specular fraction of 0.0), the simulated reflectance is 0.2011, showing a negligible deviation from the theoretical value. However, as the specular fraction increases, the simulated total reflectance systematically decreases. This deviation grows from only 1.71% at a specular fraction of 0.2 to 5.06% at a specular fraction of 0.5 and reaches a maximum underestimation of 10.55% for a purely specular surface. For the mean specular fraction of 0.3 observed on the adaxial leaf surfaces in this study, this implementation-specific behavior results in a predictable reflectance underestimation of 2.83%.

#### 3.5.4. Full-Angle URIDF Comparison at Representative Wavelengths

**Figure 12:**
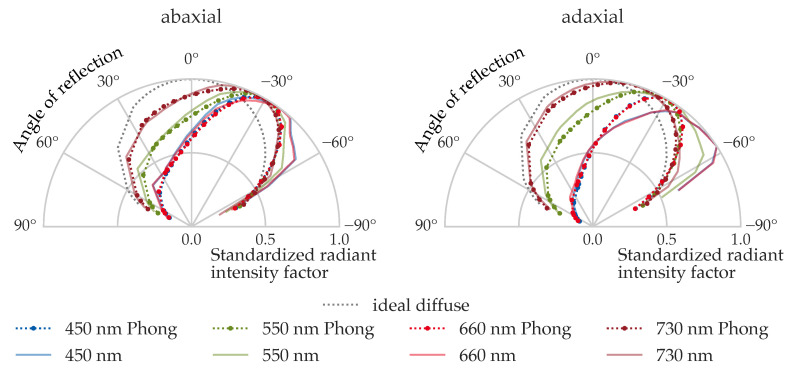
Phong URIDF (GroIMP, dotted) and measured URIDF (dashed) for a light incidence angle of 40°, in each case on the abaxial (**left**) and adaxial (**right**) leaf side of leaf order four.

To qualitatively evaluate the angular pattern fidelity of the Phong model, Figure 12 compares the measured and simulated URIDFs at four representative wavelengths (450 nm, 550 nm, 660 nm, and 730 nm) for both the abaxial and adaxial surfaces of leaf order 4, under 40° incidence.

On the abaxial side, the Phong model closely matches the shape and symmetry of the measured URIDFs. Slight differences in angular spread between wavelengths reflect increased diffuseness at 730 nm, consistent with lower absorption and higher scattering in the far-red region. At 450 nm and 660 nm, the distributions are sharper, indicating more directional reflection.

On the adaxial side, spectral dependence is more pronounced. Measured URIDFs at 550 nm and 730 nm are broader, while 450 nm and 660 nm exhibit stronger angular peaking.

This visual comparison confirms that the Phong-based URIDFs are not only numerically accurate, but also qualitatively representative of the directional reflectance behavior.

## 4. Discussion

### 4.1. Wavelength-Dependent Behavior

The observed wavelength dependence of the Phong parameters indicates that spectral variability in cucumber leaves is shaped by both pigment absorption and anatomical structure (e.g., epidermal wax layers and cuticle thickness) [21,42]. Increased variability in selected spectral regions is consistent with heterogeneity of internal structure such as mesophyll arrangement, intercellular air spaces, and local pigment density, which together modulate diffuse and specular components of reflectance.

For leaf order 3, localized deviations may reflect the above anatomical differences and measurement-related factors like alignment tolerances or small-scale surface irregularities. This combination of biological variability and measurement uncertainty should be considered when interpreting wavelength-specific patterns.

### 4.2. Angular Effects and Specular Proportion

The spectral variation of the specular proportion (Figure 8) reflects a predominance of diffuse reflection in green and far-red wavebands, consistent with strong internal scattering in the spongy mesophyll and reduced absorption in the green and far-red spectral ranges [13,42]. This predominance of the diffuse component has also been confirmed experimentally in previous studies [21,22].

At steep incidence angles, particularly at wavebands with higher specular fractions, deviations between model and measurement become pronounced. These deviations stem from the Phong model’s simplified specular term, which fails to reproduce the sharp increase in surface reflectance at grazing angles of incidence, a key characteristic of the Fresnel effect [26].

This failure to reproduce the increased reflection amplitude at 60° incidence has direct consequences for the accuracy of canopy-level simulations. The model’s underestimation of reflectance would cause a corresponding overestimation of light absorption, particularly for photosynthetically active radiation in the blue and red spectral regions where the measured effect is strongest. When propagated through a full canopy model, this systematic error could lead to inaccurate predictions of both overall photosynthetic rates and the spectral quality of the micro-light environment that governs photomorphogenesis.

### 4.3. Transferability of Parameters

The transferability tests showed that parameters fitted at one incidence angle can be applied to others with moderate accuracy. However, greater anatomical heterogeneity and directional variability between leaf surfaces may limit direct transfer to other species. Transferability will be most successful when the BRDF shape of the target species exhibits a similar balance between specular and diffuse components. Where surface curvature effects or spectral absorption features differ markedly, parameter scaling should be applied with caution. Additionally, the analysis under diffuse illumination (Table 3) showed that it could be beneficial to adjust the parameter scaling to the dominant light direction in the simulated scene to minimize differences in the total hemispherical reflection.

If the BRDF determined is to be transferred to other degrees of reflection, the corresponding data can be loaded from the Supplemental Data. Using the sub-process ‘Parameter scaling’ in Figure 6, $\rho_{m}$ and $\tau_{m}$ must be set according to own measurements and then validated with the vGRM from Supplemental Data subsequently.

### 4.4. Model Strengths and Limitations

Despite this limitation, within the 30–60° incidence range, the Phong-based BRIDF significantly reduces spectral error compared to a purely diffuse baseline (Figure 9a,b). The NRMSE of the Phong fit is consistently lower across large portions of the visible spectrum, with the highest improvements occurring outside the green and far-red regions where the specular contribution is strongest. Under 40° of light incidence, improvements over the diffuse model are significant in all spectral ranges, while they are not significant in the green and far-red regions for the angles of 60° and 30°. Under no circumstances did the Phong shader produce a larger error.

The Phong model reproduced many key spectral and angular reflectance characteristics while using only three parameters. Its compact parameterization enables integration into existing simulation environments. However, limited flexibility in modeling wavelength-dependent angular changes, especially in the specular term, constrains applicability for highly anisotropic surfaces. Compared to the diffuse model the Phong model constantly showed lower and less spectrally dependent NRMSE. At an angle of incidence of 30°, the maximum values was approximately 0.4, compared to approximately 0.8 for the diffuse model. Roth et al. [22] estimated NRMSE for different tree species between 0.07 and 0.095 using the Cook-Torrance [18,19] based microfacet models. This underlines the importance of Phong’s model as a compromise solution for increased accuracy with reduced complexity.

Our analysis revealed a systematic underestimation of total reflectance with an increasing specular fraction when using the GroIMP Phong shader under diffuse illumination. This behavior is not an error in the simulation setup but rather a practical limitation of the empirical Phong model, which, unlike physically based models, is not designed to guarantee perfect energy conservation across all conditions.

The impact of this deviation on simulation accuracy is directly related to the surface properties being modeled. For practical canopy simulations, as presented in this work, the relatively low mean specular fraction of cucumber leaves (approximately 0.3) leads to a predictable and manageable deviation of approximately 2.83%. For studies involving highly glossy surfaces or materials with a significantly higher specular component, this effect must be taken into account, as the underestimation of reflected energy can become substantial and may influence the results.

### 4.5. Practical Implications

From a practical standpoint, the Phong model offers a useful compromise between accuracy and model simplicity for light simulations. While high angular resolution BRDF measurements remain preferable for fundamental studies, wavelength-dependent Phong parameters can substantially improve realism over traditional diffuse models in greenhouse light-climate simulations. Users should be aware of the model’s limitations at steep incidence angles.

The present study demonstrates that a wavelength-dependent Phong parameterization yields a compact, simulation-ready description of cucumber leaf BRDF that captures the principal spectral and angular behaviors.

## 5. Conclusions

This study developed a method to incorporate the spectral and directional reflectance of plant leaves into virtual plant models with greater realism. We experimentally measured the spectral BRDF of cucumber leaves and subsequently derived wavelength-dependent parameters for the Phong reflectance model, which can be used directly in the GroIMP simulation environment.

The analysis demonstrated that the Phong model successfully reproduces the key angular and wavelength-dependent characteristics of leaf reflectance. Specifically, the increased diffuseness in the green and far-red spectral regions was replicated. Compared to a purely diffuse model, the Phong approach significantly reduced the per-wavelength error across broad spectral regions and for incidence angles between 30° and 60°.

In summary, this work provides a practical and computationally efficient solution to significantly improve the realism of light simulations in plant models. We provide a workflow and directly applicable parameter sets that bridge the gap between highly simplified diffuse models and data-intensive, physically complex approaches. The method, which facilitates cautious transfer to other species via scaling against hemispherical measurements, represents an advance for applications in greenhouse optimization, remote sensing and agricultural research.

## Figures and Tables

**Table 1:** Summary of the datasets acquired. The number of leaves per plant is five, with one leaf per order and multiple points per leaf. Both the adaxial and abaxial sides of each leaf are measured. * not in the range between −13° and 13° except for 0°.

Measurement Type	Points	Sides	Spectral Range	$\theta_{i}$	$\theta_{r}$
spectral reflectance (ρ(λ))	5	ab./ad.	440 to 740 nm	-	-
spectral transmittance (τ(λ))	5	ab./ad.	440 to 740 nm	-	-
URIDF(λ)–main	1	ab./ad.	440 to 740 nm	40°	−75–75° *
					interval 5°
URIDF(λ)–validation 30°	1	ab./ad.	440 to 740 nm	30°	−70–70° *
					interval 10°
URIDF(λ)–validation 60°	1	ab./ad.	440 to 740 nm	60°	−70–70° *

**Table 2:** Deviations of the reflectance determined in GroIMP at different angles of incidence of a directional light source from the reference angle at 40° in percent. The maximum measurable angle is 85°. The surface parameters are diffuse: 0.2, specular: 0.04, shininess: 0.6. Simulation was performed with $10^{7}$ rays.

	Angle of Incidence									
	0°	10°	20°	30°	40°	50°	60°	70°	80°	85°
Difference in %	1.011	0.968	0.8	0.463	0	−0.674	−1.6	−2.905	−4.674	−5.684

**Table 3:** Deviation of hemispherical reflectance as a function of specular fraction under diffuse illumination. Total theoretical reflectance was held constant at 0.20, while the specular/diffuse ratio was varied from 0% to 100%. Shininess parameter was fixed at 0.6. Simulation was performed with $10^{7}$ rays.

Parameter											
Specular fraction	0.0	0.1	0.2	0.3	0.4	0.5	0.6	0.7	0.8	0.9	1.0
Diffuse component	0.2	0.18	0.16	0.14	0.12	0.1	0.08	0.06	0.04	0.02	0.0
Specular component	0.0	0.02	0.04	0.06	0.08	0.1	0.12	0.14	0.16	0.18	0.2
Measured refl.	0.2011	0.1988	0.1966	0.1943	0.1921	0.1899	0.1877	0.1855	0.1833	0.1811	0.1789
Absolute deviation	−0.001	0.0012	0.0034	0.0057	0.0079	0.0101	0.0123	0.0145	0.0167	0.0189	0.0211
Deviation (%)	0.56	0.58	1.71	2.83	3.95	5.06	6.17	7.27	8.37	9.46	10.55

## Data Availability

The original contributions presented in this study are included in the article/Supplementary Materials. Further inquiries can be directed to the corresponding author.

## Supplementary Materials

The following supporting information can be downloaded at: https://www.mdpi.com/article/10.3390/plants14172775/s1, Supplementary File S1: Determined Phong parameters for the adaxial leaf surface (parameter_adaxial.csv); Supplementary File S2: Determined Phong parameters for the abaxial leaf surface (parameter_abaxial.csv); Supplementary File S3: GroIMP code for the virtual gonioreflectometer (GroRM.rgg).
